# ENZO: A Web Tool for Derivation and Evaluation of Kinetic Models of Enzyme Catalyzed Reactions

**DOI:** 10.1371/journal.pone.0022265

**Published:** 2011-07-19

**Authors:** Staš Bevc, Janez Konc, Jure Stojan, Milan Hodošček, Matej Penca, Matej Praprotnik, Dušanka Janežič

**Affiliations:** 1 National Institute of Chemistry, Ljubljana, Slovenia; 2 University of Primorska, Faculty of Mathematics, Natural Sciences and Information Technologies, Koper, Slovenia; 3 University of Ljubljana, Medical Faculty, Ljubljana, Slovenia; Stanford University, United States of America

## Abstract

We describe a web tool ENZO (Enzyme Kinetics), a graphical interface for building kinetic models of enzyme catalyzed reactions. ENZO automatically generates the corresponding differential equations from a stipulated enzyme reaction scheme. These differential equations are processed by a numerical solver and a regression algorithm which fits the coefficients of differential equations to experimentally observed time course curves. ENZO allows rapid evaluation of rival reaction schemes and can be used for routine tests in enzyme kinetics. It is freely available as a web tool, at http://enzo.cmm.ki.si.

## Introduction

Modern biochemical methods allow production of enzymes in large amounts and in many variations, and physical methods provide valuable information concerning their functional characterization. Among these, kinetic methods which monitor the time course of an enzyme-catalyzed reaction are the oldest and most widely used. The Michaelis-Menten reaction mechanism was proposed almost a century ago to describe the reaction of enzymes with their substrates [1] and it is still usually the first candidate that is tested on each newly examined enzyme. New experimental techniques have revealed however that enzymes typically adjust their activity according to environmental demands and this leads to substantial deviations from classical hyperbolic kinetics [2]. Thus, a common simplification used in enzyme kinetics is that initial rates are analyzed rather than the complete time course of the reaction under investigation. This leads to enormous loss of information [3]. Moreover, the analytical solution of ordinary differential equations cannot readily be derived, even for a simple Michaelis-Menten single intermediate reaction mechanism [4], let alone for the systems including inhibitors or which are allosterically regulated.

The differential equations corresponding to the Michaelis-Menten scheme
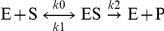
are:
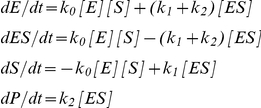
where E represents the free enzyme, S the substrate, ES the Michaelis complex and P the product; *k_0_* is a second order rate constant, and *k_1_* and *k_2_* are first order rate constants.

If we specify that the free enzyme E and the complex ES are in equilibrium (defined with the equilibrium constant *K*), the following conservation equation is applicable
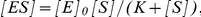
and the above system of ordinary differential equations simplifies to
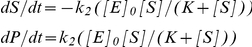
There are various numerical methods to solve these differential equations and establish the time course of each participating species (see e.g., [5], [6]). However, these numerical methods should be carefully selected and implemented for the evaluation of reaction mechanisms and relevant parameters in biological systems [7], [8], i.e., a numerical solver should be linked to a regression analysis algorithm. Computational tools that meet these criteria are uncommon and not user-friendly. Aside from the details of the algorithm, the major drawback is the labor-intensive preparation and implementation of differential equations specific to the system, a procedure which must be repeated for every new reaction mechanism that is to be tested. To overcome this difficulty, we have adopted an appropriate fitting numerical solver [8] and linked it to an interactive graphical web user interface.

In this paper we present the web tool ENZO, which runs on a freely accessible public web server. ENZO automatically generates differential equations from proposed enzyme kinetics reaction schemes and fits the coefficients of differential equations to concentration progress curves. Thus, one can quickly evaluate several different reaction schemes and find the one that is optimal for the enzyme reaction at hand.

## Methods

Among biochemical methods, kinetic methods are the most widely used to provide us with information how fast and in what sequence enzymes associate or dissociate with their cofactors or other enzymes and process their substrates. Initially, we assume that multiple such pathways are possible and therefore we have to test each and every one by building a corresponding kinetic model and determine how well the latter agrees with experimental data in the form of progress curves. To this end, we have developed a web interface that automates this cumbersome time consuming task by presenting enzyme reactions as graphical diagrams. This enables quick testing and evaluation of different kinetic models. The final kinetic model, which agrees most closely to experimental data, allows us to understand the steps of a given enzyme reaction, which in turn leads to a better understanding of enzyme functions.

### ENZO web tool

**Figure 1:**
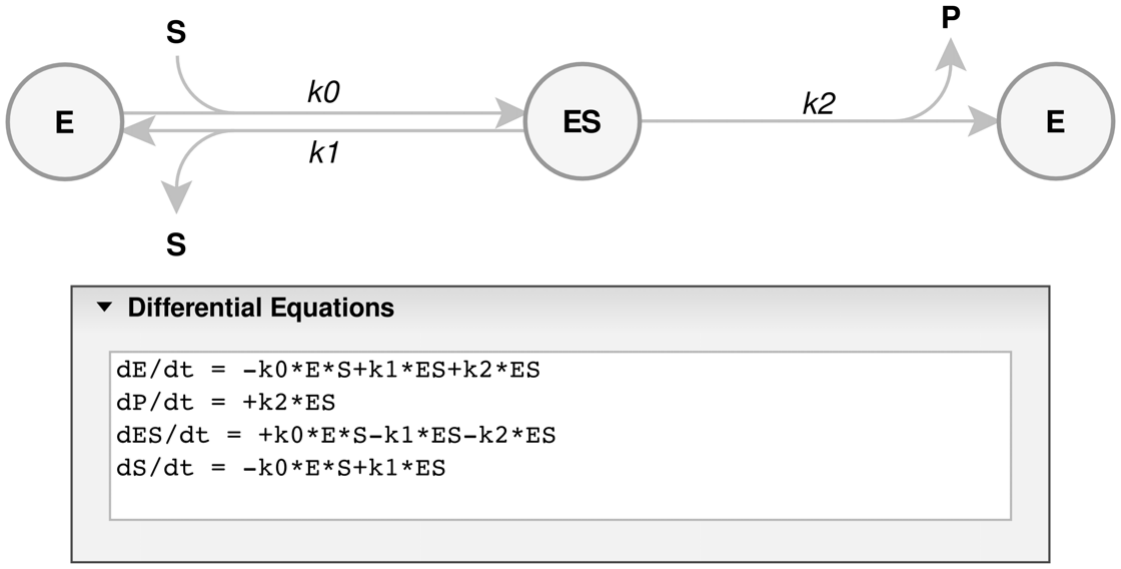
Michaelis-Menten reaction scheme. E is the free enzyme, S the substrate, ES the Michaelis complex and P the product; *k_0_* is a second order and *k_1_* and *k_2_* are first order rate constants, respectively. The differential equations were automatically generated from the drawn reaction scheme by ENZO.

To analyze time resolved kinetic data one must develop solutions, either analytical or numerical, to a system of ordinary differential equations which describe the relevant reaction mechanism. In principle, for equations assumed to be first order or pseudo first order, an analytical solution can be derived. However, if the course of a reaction involving multiple intermediates is followed until its completion, or if it is coupled with a second reaction, the depletion of reactants becomes significant and the simplifications are no longer applicable. To avoid manual development of the differential equations from a proposed reaction scheme, ENZO generates the relevant differential equations from user-defined schemes. The equations are generated using recursive rules, and the process, an example of which is shown in Figure 1 for the Michaelis-Menten scheme, is independent of the complexity of the reaction scheme.

### Implementation: Software and Hardware

It is in many cases possible with modern web technologies to create applications that are as functional as desktop applications. Compared to desktop programs, web applications actually have certain advantages, one of which is that they do not need an installation process and typically function independently of one's computer operating system. This makes them immediately available to anyone with a broadband Internet connection.

**Figure 2:**
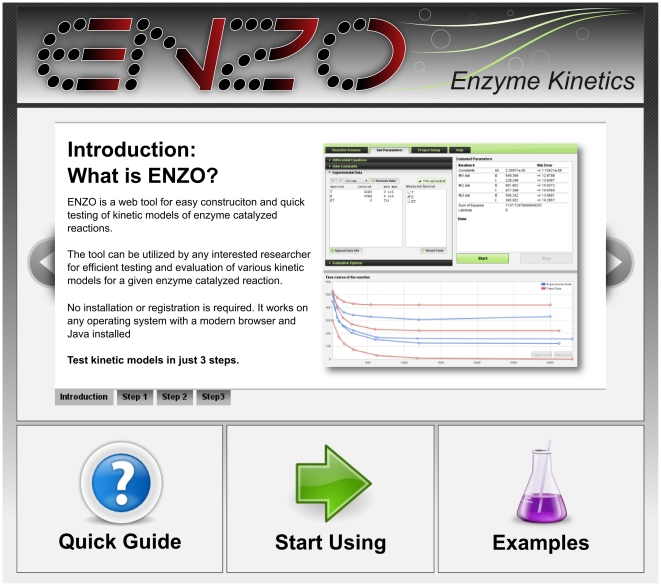
ENZO URL: http://enzo.cmm.ki.si **.** The ENZO web page provides a short introduction and links to a quick guide, examples and ENZO tool.

ENZO is an interactive web application developed using web programming technology. The ENZO web page, displayed in Figure 2, is at http://enzo.cmm.ki.si. ENZO has client-server architecture. The client-side code runs in one's web browser and is responsible for generating a user interface. The reaction scheme is programmed in Java (James Gosling, Sun Microsystems, Oracle, http://www.oracle.com/technetwork/java/index.html) as an applet, the dynamic parts of the user interface are programmed in JavaScript using the jQuery library (J. Resig, http://jquery.com). Both are placed in HTML which also renders the non-dynamic parts of the user interface. jQuery is used for the communication with the server, and communication is accomplished with an XMLHttpRequest object (World Wide Web Consortium, http://www.w3.org/TR/XMLHttpRequest/).

This technique, also known as Ajax, allows web browsers to request data from the server and display or manipulate the server's response without reloading the web page. In traditional web programming, users must wait for the data to be processed by the server and pages reloaded in order to display new information. In ENZO, the integration with the server is seamless in that the user cannot distinguish between the processing being done by the client and that done by the server. This creates the impression that ENZO is a stand-alone program, but at the same time it retains the benefits of web applications.

The server side consists of a computer running the Apache web server and a scheduler, and several computers running numerical solvers with GNU/Linux as their operating system. The web server code is programmed in the PHP programming language and the numerical solvers are programmed in C. When an evaluation request is made, PHP interprets it and writes it in a queue file. The scheduler reads the queue file and dispatches jobs to computers running numerical solvers. This ensures that every evaluation has enough resources available and that multiple evaluations can be made simultaneously. In the event that all the computers are busy, the client requests simply wait in queue until a computer becomes free. The evaluation output is written to a temporary file on the web server, read by PHP and returned to the client and displayed in one's web browser.

Each ENZO computer has multiple multicore processors; curve fitting is run in parallel on multiple processor cores and as a result, multiple curves can be fitted rapidly. For example, fitting the kinetic model presented in Example 3 (see Results section) requires about 1 min. The computation time however depends on whether or not convergence is reached.

### Input

ENZO evaluates proposed kinetic models by fitting the parameters of the corresponding differential equations to experimental data, termed “progress curves” or “time courses”. Progress curves can represent the changes in absorbance, which is linearly correlated with concentration of detectable reaction species (i.e., substrates, products, intermediates), at discrete time intervals. Experimental data is provided as a text file with two columns separated by space or tab. The second column carries the concentration of species at the time noted in the first column. Each row thus represents a measurement in time and each file represents a single progress curve. Preparation of the parameters that define the initial conditions and estimated rate constants is described below and in detailed instructions on the web server pages.

#### i) Draw Reaction Scheme

**Figure 3:**
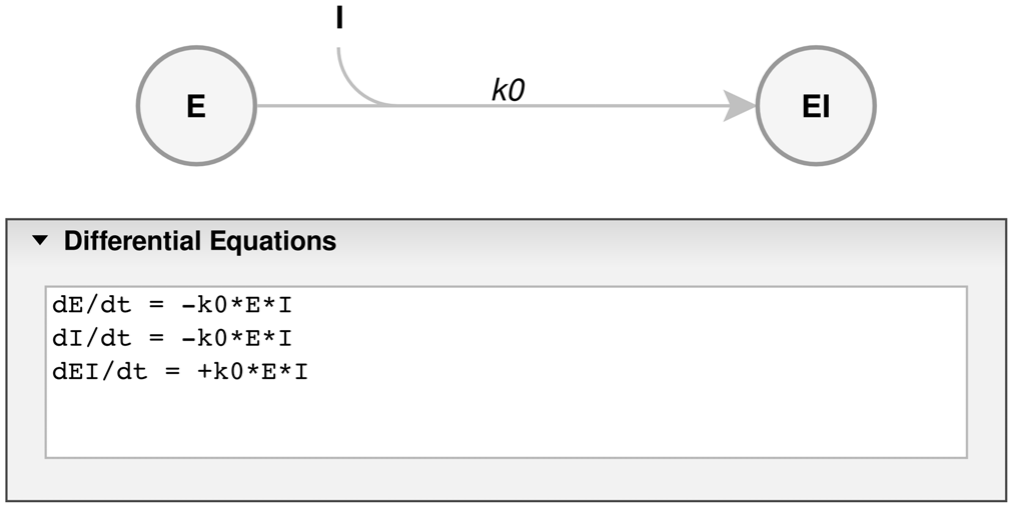
Enzyme titration reaction scheme. E is the *Torpedo californica* acetylcholinesterase enzyme, I the m-(N,N,N-trimethylammonio) trifluoroacetophenone (TMTFA) inhibitor, and EI their complex; *k_0_* is a second order association rate constant. The reaction was drawn using the *Reaction Scheme* tab of ENZO.

ENZO has a graphical user interface that allows definition and drawing of complex reaction schemes. One draws the kinetic scheme in the *Reaction Scheme* tab on the ENZO page, where the enzyme reactions are represented as nodes and arrows. Text labels within the nodes represent reaction species, e.g., enzymes, substrates, products, or cofactors; labels above or below the arrows represent kinetic constants as in, e.g., Figures 1 and 3. Each such scheme defines a unique set of differential equations, which are automatically generated from the constructed scheme.

#### ii) Set Parameters

**Figure 4:**
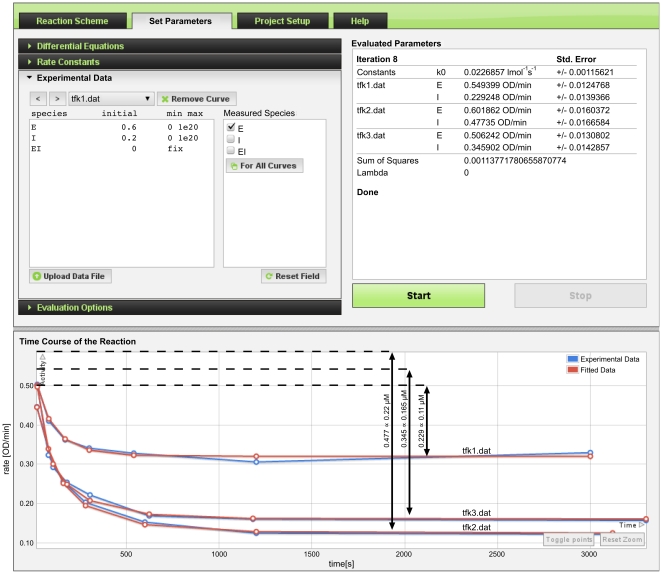
Converged results of parameter fitting for enzyme active site titration experiment. Initial concentrations of enzyme E and inhibitor I for progress curve files tfk1.dat, tfk2.dat, tfk3.dat (*Experimental Data* panel shows tfk1.dat) are fitted in the interval of [0, 10^20^]; the initial concentration of EI is zero and fixed; the checkbox “E” is checked under *Measured Species*, which signifies that E is the measured quantity and the progress curves below represent the time course of its residual activity. The respective units of the residual activity in the Y-axis are OD/min and the units of time in X-axis are seconds. Fitted rate constant *k_0_* and initial values of E and I at three different concentrations of I are displayed under the *Evaluated Parameters* in the upper right corner panel, the experimental progress curves are blue and the fitted curves are red as shown in *Time Course of the Reaction* chart at the bottom panel of the screen. The arrows mark the difference between the inital value and the plateau.

Once the reaction scheme is established, the *Set Parameters* tab is used to define the initial conditions and estimates of rate constants with rational limits. The measured time course data are then uploaded as progress curves. One can select and upload multiple progress curves or alternatively, it is possible to upload a compressed .zip file containing multiple progress curves. When uploaded, these are shown as blue curves in the *Time Course of the Reaction* chart (e.g., Figure 4 bottom). The initial concentration and the identity of the measured species corresponding to each uploaded data file are defined. The initial values of rate constants and species concentrations can also be set to a constant value, in which case the server will perform a first approximation without any fitting of the proposed kinetic model.

#### iii) Evaluation of Parameters

When an evaluation is started, information is delivered to the solver which starts an iterative evaluation and displays regression results for each iterative step. It is possible to evaluate simultaneously experimental progress curves which describe various reaction intermediates, if the information is available.

### Output

The output of ENZO is a set of fitted kinetic parameters that best describe the concordance between theoretical curves of the proposed kinetic model and the experimental kinetic data. If the convergence is reached, the proposed model is considered to be plausible and confirmed; otherwise the user is encouraged to repeat the calculation with a different set of initial parameters and species concentrations. If this fails to result in convergence, modification of the proposed kinetic model and repetition of the process will be necessary.

## Results

The applicability and efficiency of ENZO have been assessed on three different kinetic models in each of three different actual enzyme-kinetics scenarios, which are described in detail here. These examples are also available for review on the ENZO web page http://enzo.cmm.ki.si.

### Example 1: Active site concentration and enzyme activity correlation

A common task in enzymology is determination of the concentration of species containing an active site. In the kinetic characterization of a new enzyme, active site titration allows estimation of reaction mechanism parameters, such as the turnover number. We titrated the active site of *Torpedo californica* acetylcholinesterase (TcAChE) using approximately equimolar concentrations of m-(N,N,N-trimethylammonio)trifluoroaceto-phenone (TMTFA), a transition state analogue with affinities reported [9, PDB code 1AMN] to be in the atomolar range. After various incubation times aliquots were diluted by a factor of 300 and the residual enzyme activity in the presence of 0.5 mM acetylthiocholine (ATCh) was measured by a standard method [10]. For a reliable determination of active site concentration, we measured three curves with different initial enzyme and titrant concentrations. The reaction between the TcAChE and TMTFA is theoretically reversible, but due to the high TMTFA affinity for the active site, it can be regarded as essentially irreversible, and written as in Figure 3 prepared with ENZO.

**Figure 5:**
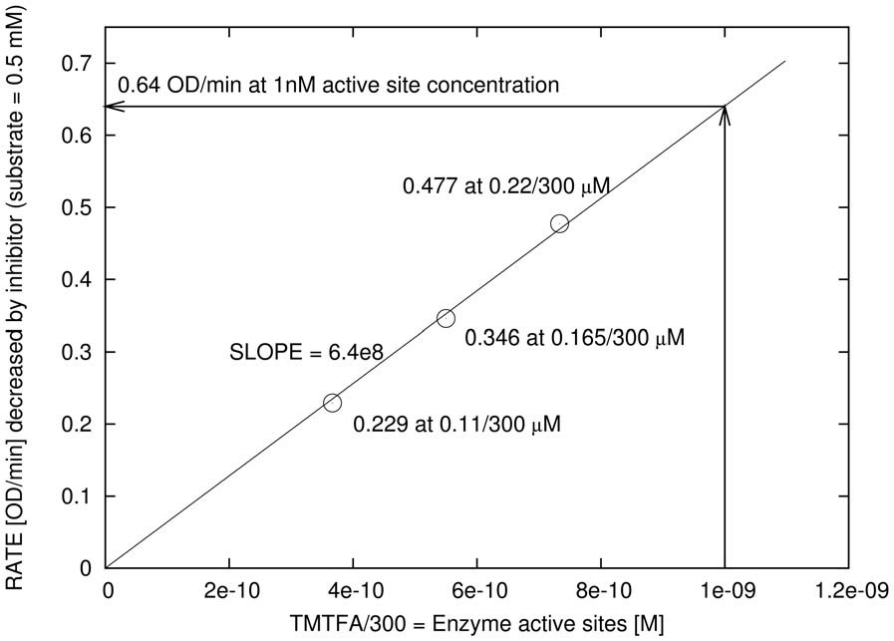
Normalization curve for the determination of active site concentration from enzyme activity. X-axis represents the concentration of added TMTFA corresponding to actual enzyme concentrations after 300 times delution. Y-axis represents the difference between the initial enzyme activity and the activity at plateau caused by the presence of experimental TMTFA concentration. The concentration of the substrate in all activity determinations was 0.5 mM.

The parameters, i.e., the rate constant *k*_0_ and the initial concentrations [E] and [I] were fitted to the experimental progress curves by the numerical solver with non-linear regression [8] implemented in ENZO. From the fitted initial TMTFA optical density (OD) values of 0.229, 0.346, 0.477 (see *Evaluated Parameters* in Figure 4) corresponding to three known concentrations of 0.11 µM, 0.165 µM and 0.22 µM of added TMTFA, respectively, it follows (see Figure 5 displaying the normalization curve) that if adding 0.5 mM ATCh and measuring the enzyme activity of 0.640 OD/min, then the corresponding concentration of active sites of TcAChE is 1 nM. We report here, for the first time, the second order rate constant for the binding of TMTFA to TcAChE: 47228+/−2407 M^−1^ s^−1^ as computed by ENZO.

The corresponding analytical solution for this example is:
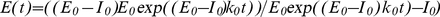
and the determined second order rate constant is 47225+/−2407 M^−1^ s^−1^ which totally agrees with the ENZO numerical solution.

### Example 2: Autoactivation of Procathepsin B

Some enzymes are produced as inactive precursors, called zymogens, and enzyme activation is achieved by chain shortening, a biochemical modification often performed by the active enzyme itself. The time course of such autoactivation typically starts with a lag period, followed by a rapid burst, and ends in a plateau when all zymogen has been converted into the active form.

**Figure 6:**
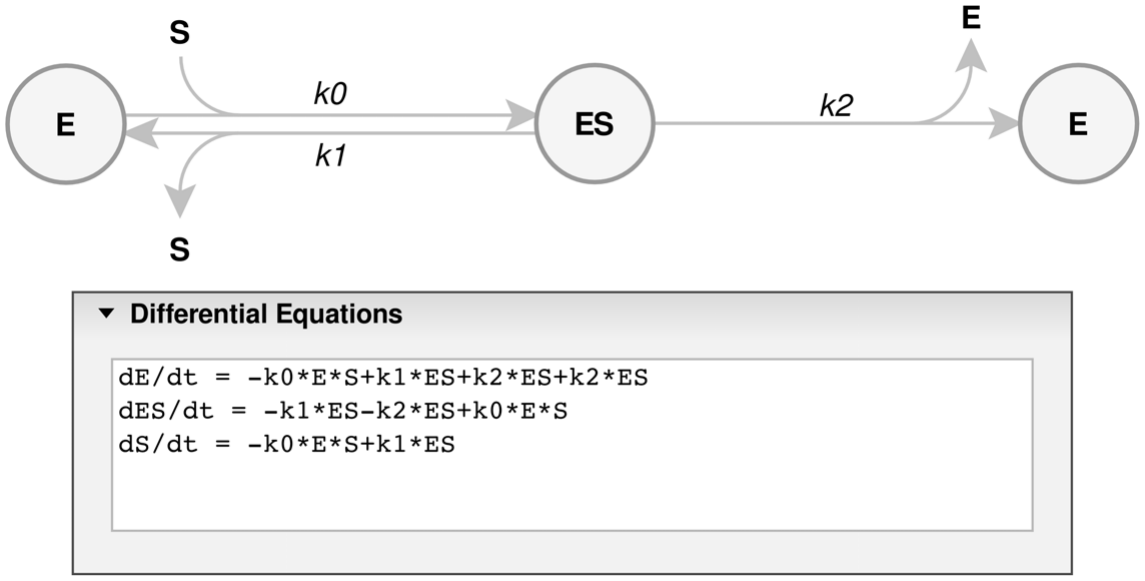
Reaction scheme for autoactivation of procathepsin B. E stands for free active enzyme, S for enzyme precursor and ES is the complex. Note the stoichiometry of the reaction in the differential equations.

We have generated a model of this autoactivation process as presented in Figure 6, and as originally proposed in reference [11]. ENZO's solution obtained under non-equilibrium assumptions is thus comparable with the corresponding solution in reference [11], which was, however, conducted under mixed equilibrium and steady state assumptions. The latter is taken as a benchmark to validate the ENZO performance. Recently, alternative mechanism for this reaction, which assumes that the procathepsin itself is active, has been proposed [12], but is not tested here.

**Figure 7:**
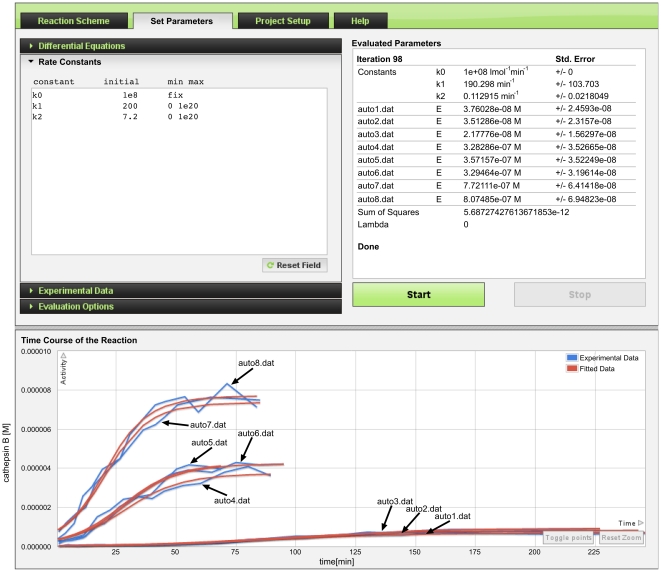
Autoactivation of procathepsin B. The data originally presented in [11] were used. The total amount of protein was determined by Pace et al. [13] and used as a fixed value for each substrate/precursor concentration. The initial values of ES is zero and E is fitted in the interval [0, 1]. The initial value of rate constant *k_0_* is set to diffusion rate value of 10^8^ M^−1^ min^−1^ and fixed, while initial values of *k_1_* and *k_2_* are 200 min^−1^ and 7.2 min^−1^ respectively, and fitted in the interval of [0, 10^20^]. The sum of free active enzyme (E) and the instantaneusly dissociated complex (ES) is the measured species. Y-axis represents the concentration of cathepsin B in molar concentration and the X-axis represents time in minutes. The final estimated values of rate constants and initial concentrations of active enzyme portion for each individual curve are displayed under the *Evaluated Parameters*.

The numerical results derived by ENZO are displayed as the red curves in Figure 7 and are in good agreement with the blue experimental curves. Moreover, the fitted values of kinetic constants match those reported in [11]. The minor differences stem from the different treatments employed, i.e., non-equilibrium (Ks = *k_1_*/*k_0_* = 1.9+/−1.0 µM, *k_cat_* = *k_2_* = 0.11+/−0.02 min−1) vs. mixed equilibrium and steady state assumptions (Ks = 2.1 µM, *k_cat_* = 0.12 min^−1^), and from the step size used in ENZO numerical integration. The discrepancies in the initial concentrations of active enzyme (E values in Figure 7) between the different progress curves are a consequence of autoactivation of pro-cathepsin B prior to the start of the experiment. Even if total enzyme concentrations were equal in all experiments, the inital concentration of active enzyme may vary because the ratio depends on the age of the sample. If the same batch was used, the same ratio would be expected. To evaluate the actual ratio, we fitted the initial active concentrations for each individual measurement.

### Example 3: Cholinesterase reaction with butyrylthiocholine

Cholinesterases hydrolyze choline esters effectively and are strongly inhibited by 10 µM concentrations of physostigmine [2]. Due to specific structural properties, such as a buried active site, a peripheral substrate binding site and a backdoor channel, cholinesterases are highly efficient and specific, but in general, they do not obey hyperbolic Michaelis-Menten kinetics in their reactions with substrates. They are active over a very wide concentration range of substrate, are activated at intermediate concentrations and strongly inhibited at very high substrate concentrations. Kinetics of this sort has been explained in terms of the following molecular events:

1. the substrate first binds to the peripheral anionic site (PAS), with the affinity of *k_1_*/*k_0_* at the active site entrance
2. then it descends and reaches the catalytic anionic site (CAS), with partition coefficient *k_3_*/*k_2_*, where
3. it is hydrolyzed according to a covalent catalysis mechanism with acylation rate constant *k_4_* and deacylation rate constant *k_5_*.
4. alternatively, if the substrate molecule descends to the CAS of acylated enzyme (EA, with the partition coefficient *k_13_*/*k_12_*), it would block the deacylation process.

**Figure 8:**
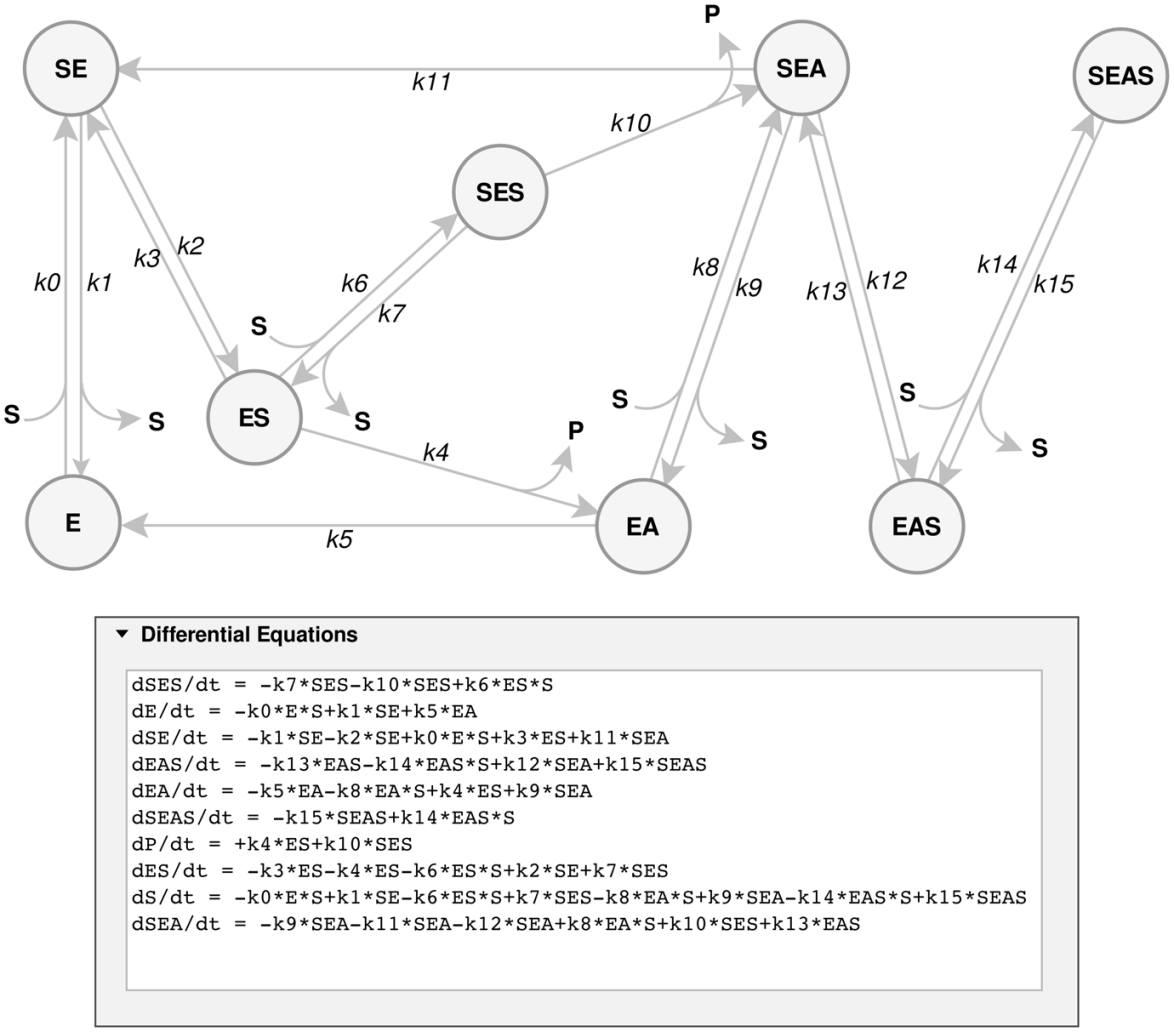
Cholinesterase reaction with a substrate. The reaction scheme and the corresponding differential equations were created by ENZO. Substrate (butyrylthiocholine) bound to the peripheral anionic site is denoted by “S” on the left of the label name (e.g., SE, SES, SEA, SEAS). When bound to the catalytic anionic site, the “S” is placed on the right of the name (e.g., ES, SES, EAS SEAS). Covalent acyl-enzyme is represented by EA and P is the first product (thiocholine) released upon enzyme acylation. The acyl group is denoted by A.

The active site in cholinesterases is relatively large and a second substrate molecule can bind to the PAS with the affinity of *k_7_*/*k_6_* = *k_9_*/*k_8_* = *k_15_*/*k_14_* at different times before the turnover of the first substrate is completed at the CAS [14]. In Figure 8, substrate bound to the PAS is denoted by “S” on the left of E e.g., SE. When bound to the CAS, the “S” is placed on the right, e.g., ES. Covalent acyl-enzyme is represented by EA and P is the first product (thiocholine) released upon enzyme acylation. The release of the acyl group (A) is unimportant, and is therefore omitted. In the case of butyrylcholinesterase (BChEs) a second substrate molecule, bound to PAS, enhances both the acylation as well as the deacylation steps, shown in Figure 8 as SES→SEA and SEA→SE, respectively. Thus *k_11_*>*k_5_* and *k_10_*>*k_4_*. All this information can be easily drawn by ENZO as the comprehensive reaction scheme shown in Figure 8 [15] and subsequently the experimental data can be evaluated by fitting the parameters of the corresponding differential equations.

**Figure 9:**
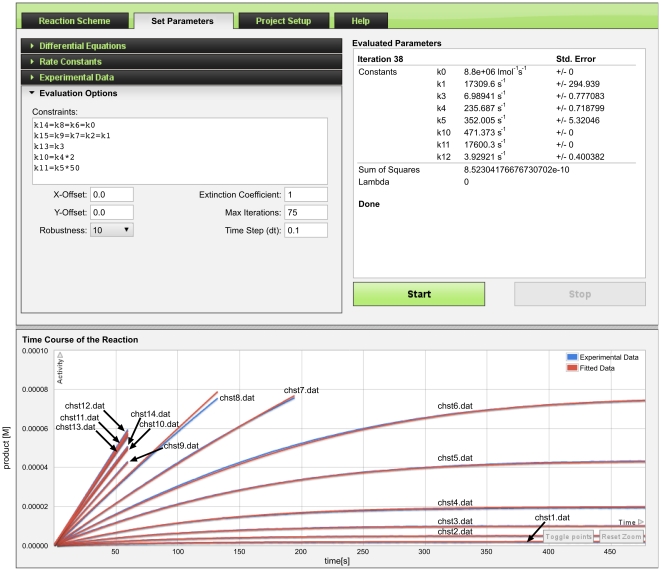
Converged results of parameter fitting for inhibition of cholinesterase. Fitted rate constants *k_1_*, *k_3_*, *k_4_*, *k_5_*, *k_10_*, *k_11_*, *k_12_* are displayed under the Evaluated Parameters. *k_0_* is fixed at 8.8·10^6^ s^−1^. ENZO allows, if necessary, for constraining several rate constants, thus simplifying the fitting and speeding up the evaluation process (*k_14_* = *k_8_* = *k_6_* = *k_0_*, *k_15_* = *k_9_* = *k_7_* = *k_2_* = *k_1_*, *k_13_* = *k_3_*, *k_10_* = *2k_4_*, *k_11_* = *50k_5_*, where *k_14_*, *k_8_*, *k_6_*, *k_0_* are second order while others are first order rate constants). For all progress curves, the initial concentrations of SES, SE, EAS, ES, SEAS, P, ES, SEA are fixed to zero and not fitted. E is fixed to 2.6 nM as independently determined by active site titration. For the progress curves chst1.dat to chst14.dat, S is fixed to 2 µM, 5 µM, 10 µM, 20 µM, 4.35 µM, 7.63 µM, 0.16 mM, 0.5 mM, 1 mM, 2 mM, 5 mM, 10 mM, 20 mM and 5 mM respectively. P is the measured species. Y-axis shows the product in molar concentration and X-axis shows the time in seconds.

In the conventional colorimetric cholinesterase detection method [10], a thio analogue of a substrate is used and the first product (P) is thiocholine. Its reaction with the thio reagent dithio-bis-nitrobenzoic acid (DTNB) yields a yellow color in stochiometric proportion with the released product. The time course of production of the yellow color in the butyrylthiocholine hydrolysis by horse BChE was measured at 14 different initial substrate concentrations, from 2 µmol to 50 mM in a 20 mM phosphate buffer solution, at pH 7 and 25°C. To avoid further complications resulting from newly formed products and/or DTNB depletion from its initial 0.6 mM concentration, the reaction was followed until a 60–80 µM concentration of thiocholine appeared. Under these conditions, all substrate is hydrolyzed at low concentrations giving rise to the plateaus in five of the curves and mainly linear product formation occurs at all other concentrations as seen in Figure 9.

The added reactant concentrations along with rational first estimates of kinetic constants must be provided prior the ENZO evaluation. When a complex reaction scheme is analyzed, there are a large number of relevant parameters and in practice it is impossible to evaluate them independently. To proceed, several of them can be defined as fixed or equal in the ENZO constraints (*Evaluation Options* in Figure 9), thus substantially reducing the number of unknowns. In our Example 3 we set *k_10_* = 50*k_4_* and *k_11_* = 2*k_5_* since it is known that BChE is substantially activated at high substrate concentrations [16] and that several peripheral and active site ligands accelerate its covalent modification [17]. Such simplifications are necessary when intermediates, whether theoretically predicted or mechanistically obligatory, increase the number of unknowns beyond the reach of the available experimental information.

## Discussion

The basic experiment in enzyme kinetics is a measurement of the time course of reactants and/or products concentrations. Analysis of such data is a complex task, and consequently, explicit analytical solutions can be reached only for the part of the curve where pseudo first order assumptions are valid. In cases in which the reaction proceeds under second order conditions, an analytical solution can only be reached for a single step mechanism, as in Example 1, above. Reactions catalyzed by enzymes however, often consist of several consecutive steps and are usually a mixture of elementary first and second order steps. Of the many approaches devised to overcome this problem, the most widely used is analysis of the initial rates. This has several advantages, but uses only a small proportion of the information in progress curves, hence a numerical approach to analysis of progress curves would be preferred.

There have been several attempts to overcome this problem. The basic task is the use of a numerical algorithm to solve a system of ordinary differential equations. This affords the time course of concentration changes of all reactants and products in a given reaction scheme. Thus far it has been necessary to write the differential equations specific to the scheme – a cumbersome task which must be repeated for each new mechanistic variant when complex and branched mechanisms are tested. ENZO facilitates precisely this step and hence allows rapid discrimination between several putative reaction schemes and determination of a reaction mechanism along with relevant kinetic parameters, as demonstrated in the numerical examples provided here. The applicability of ENZO is not restricted to enzyme kinetics; it can be applied to any kinetic system, such as pharmacokinetics, that can be modeled by a set of ordinary differential equations.
